# Parental decision-making for circumcision of children aged 6–12 years in China: trends, influencing factors and the role of the media

**DOI:** 10.3389/fruro.2025.1730269

**Published:** 2025-12-09

**Authors:** Yue Wu, Yi Wei, Xiang Liu

**Affiliations:** 1 School of Journalism and Communication, Sichuan International Studies University, Chongqing, China; 2 Department of Urology, Children's Hospital of Chongqing Medical University, Chongqing, China; 3 Department of Pediatric surgery, YiBin Hospital Affiliated to Children‘s Hospital of Chongqing Medical University, Sichuan, China

**Keywords:** circumcision, phimosis, children’s health, parental decision-making, internet influence, healthcare resource allocation

## Abstract

**Objective:**

This study investigates the factors influencing Chinese parents’ decision to pursue circumcision for their children aged 6–12 years and analyzes the role of the Internet and media in the popularization of related scientific knowledge.

**Methods:**

Based on retrospective data from the National Children’s Regional Medical Single Center from 2015 to 2024, along with a questionnaire survey and literature analysis.

**Results:**

The study found a significant increase in clinic visits (p = 0.000, r = 0.967) and circumcision procedures (p = 0.003, r = 0.831) among children with phimosis over the past decade. Circumcision is no longer entirely dependent on medical professional advice, but is influenced by a wider range of informational and sociocultural factors. Parents’ trust in online medical information (OR = 6.054, 95% CI 1.027-35.683, p = 0.047) and prolonged exposure to media content recommending surgery (OR = 7.500, 95% CI 1.481-37.974, p = 0.015) were independent risk factors contributing to their decision. In contrast, fear of surgical risk (OR = 0.239, 95% CI 0.059-0.965, p = 0.044), anesthesia, high costs, and long recovery times were key deterrents. Further validation of these independent risk factors revealed that internet and social media platforms (e.g., Baidu, Xiao hongshu, and Shake) had a significant impact on parental decision-making through algorithmic recommendations and emotional content, creating an “opinion dominance effect.”

**Conclusion:**

The study reveals the complexity of parental decision-making and emphasizes the need to enhance the dissemination of scientific information to mitigate the impact of misleading content on medical decision-making.

## 1 Introduction

Phimosis is a physiologic or pathologic condition in which the foreskin fails to retract, thereby preventing exposure of the glans penis. Almost all male newborns are born with physiologic prepuce, which gradually detaches from the glans over time and eventually retracts spontaneously without the need for medical intervention (1). However, in clinical practice in China, many parents choose to circumcise their children despite the lack of clear surgical indications - an issue that has drawn considerable attention from both the medical community and scholars in health communication.

Medical studies have identified the primary indications for circumcision to include pathological phimosis (i.e., an extremely narrow opening of the foreskin that impairs urination or leads to recurrent infections), recurrent episodes of balanitis or urinary tract infections, and phimosis that interferes with sexual activity. However, the large number of pediatric circumcision procedures in clinical settings suggests that many parents’ decisions are not strictly based on medical indications and may be influenced by non-medical factors. Such unnecessary surgical interventions not only increase the surgical risks for scarring, bleeding, infection, and cosmetic complications (2) but may also result in inefficient use of healthcare resources, and even impose heavier financial and psychological burdens.

Circumcision rates in China appear to be on the rise in recent years, despite the absence of religious or cultural mandate. This trend may be driven by a combination of factors, including parental perceptions of child health, medical advice, socio-cultural beliefs, peer influence, and the dissemination of information through the Internet and mass media. In particular, against the backdrop of the popularity of new media and social networking platforms, there have been profound changes in the way parents access health information. The dissemination of medical information is no longer limited to professional medical consultations, but is influenced by multiple media sources such as short videos, popular science articles, and social discussions. However, the accuracy, authority and objectivity of the information vary, and some misleading content may exaggerate the medical necessity of circumcision, reinforce parental anxiety and thus contribute to a higher incidence of the procedure. Furthermore, the widespread availability and promotion of circumcision as a safe, minor procedure performed by healthcare professionals in clinical settings may itself be a contributing factor. The enhanced safety profile and minimal invasiveness of modern surgical techniques are likely to alleviate parental concerns to some extent, thereby increasing their willingness to consent to the procedure even in the absence of strong medical indications.

This study aims to clarify the recent trends in circumcision rates in China, systematically analyze the key factors influencing parental decision-making, and ultimately explore the role of the Internet and mass media in disseminating information about circumcision, with particular attention to the potential impact of misinformation. Meanwhile, this study seeks to provide a scientific basis for the formulation of public health policies, to promote rational and evidence-based medical decisions, to reduce unnecessary surgical interventions, to optimize the allocation of medical resources, and to enhance public health literacy.

## 2 Methods

This study used multidimensional data sources and comprehensive analysis methods to systematically assess the key factors influencing parental decisions regarding circumcision and to explore the role of the Internet and media in health information dissemination. The research design included a clinical data review, questionnaire survey, internet data analysis and statistical modeling to ensure the scientific validity and applicability of the findings.

### 2.1 Clinical data review

In this study, we retrospectively analyzed data from children diagnosed with phimosis, redundant prepuce, or incarcerated phimosis at the Children’s Hospital of Chongqing Medical University between January 2015 and September 2024. Patients who underwent circumcision as part of surgical management for other urological conditions (e.g., posterior urethral valves, vesicoureteral reflux, hypospadias) were excluded from the analysis, as the decision-making rationale in these cases differs fundamentally. The dataset included information on the year of visit, diagnoses, outpatient volume, and number of circumcision procedures performed. All data were anonymized to protect patient privacy and were exempted from ethical approval according to relevant regulations.

### 2.2 Questionnaire survey

To further understand the sources of information and factors influencing parental decision-making, a questionnaire survey was conducted between July and September 2024 among 56 parents of pediatric patients at the outpatient clinic of Children’s Hospital of Chongqing Medical University. The survey collected data on the children’s age, guardian’s education, and economic status, access to health information (including doctor’s advice, social media, and influence of family and friends) and parental knowledge and attitudes toward circumcision, including perceptions of its necessity, awareness of potential risks, and expectations regarding postoperative outcomes. The study also analyzed the psychological mechanisms behind parental decision-making and evaluated the role of external information sources in this process. Although 120 questionnaires were distributed, the instrument was designed with an extensive set of items to enable a comprehensive assessment of the psychological mechanisms underlying parental decision-making and the role of external information sources. This resulted in a final yield of 56 valid responses, representing a completion rate of 46.67% (56/120). This relatively low response rate and the consequent modest sample size inevitably constrain the generalizability of the findings and may affect the stability of the regression models.

### 2.3 Online data analysis

To examine how the Internet and media environment shape public perceptions of circumcision, this study employed two web-based data analysis methods: (1) To analyze the academic literature, we searched Baidu Scholar, Wipo, Wanfang and other databases for studies related to circumcision and phimosis. Research hotspots and trends were identified, and Cite Space software was used to construct a knowledge map to further analyze these trends; (2) Social media data mining was conducted by crawling 20 related posts with highly interactive posts related to circumcision on Xiaohongshu (42,574 total comments). Natural language processing (NLP) techniques were applied to perform sentiment analysis to assess public emotional tendencies and the mechanism of public opinion influence. High-frequency opinions on circumcision were identified across social platforms, and the main topics and discourse structures in the dissemination of information were analyzed.

### 2.4 Statistical analysis

All data were analyzed using SPSS 27.0 statistical software, with the significance level set at p < 0.05.

For categorical variables, the chi-square test (χ²) was used to compare the distribution of willingness to undergo surgery, sources of information and factors influencing decision-making in different populations.

For continuous variables, independent samples t-tests or Mann-Whitney U-tests were used to compare group differences in surgery willingness scores and the parent health perception index.

To identify independent risk factors influencing surgical decision-making, binary logistic regression was conducted, with age, education, and physician recommendation as key predictors.

## 3 Results

### 3.1 Trends in circumcision

**Figure 1:**
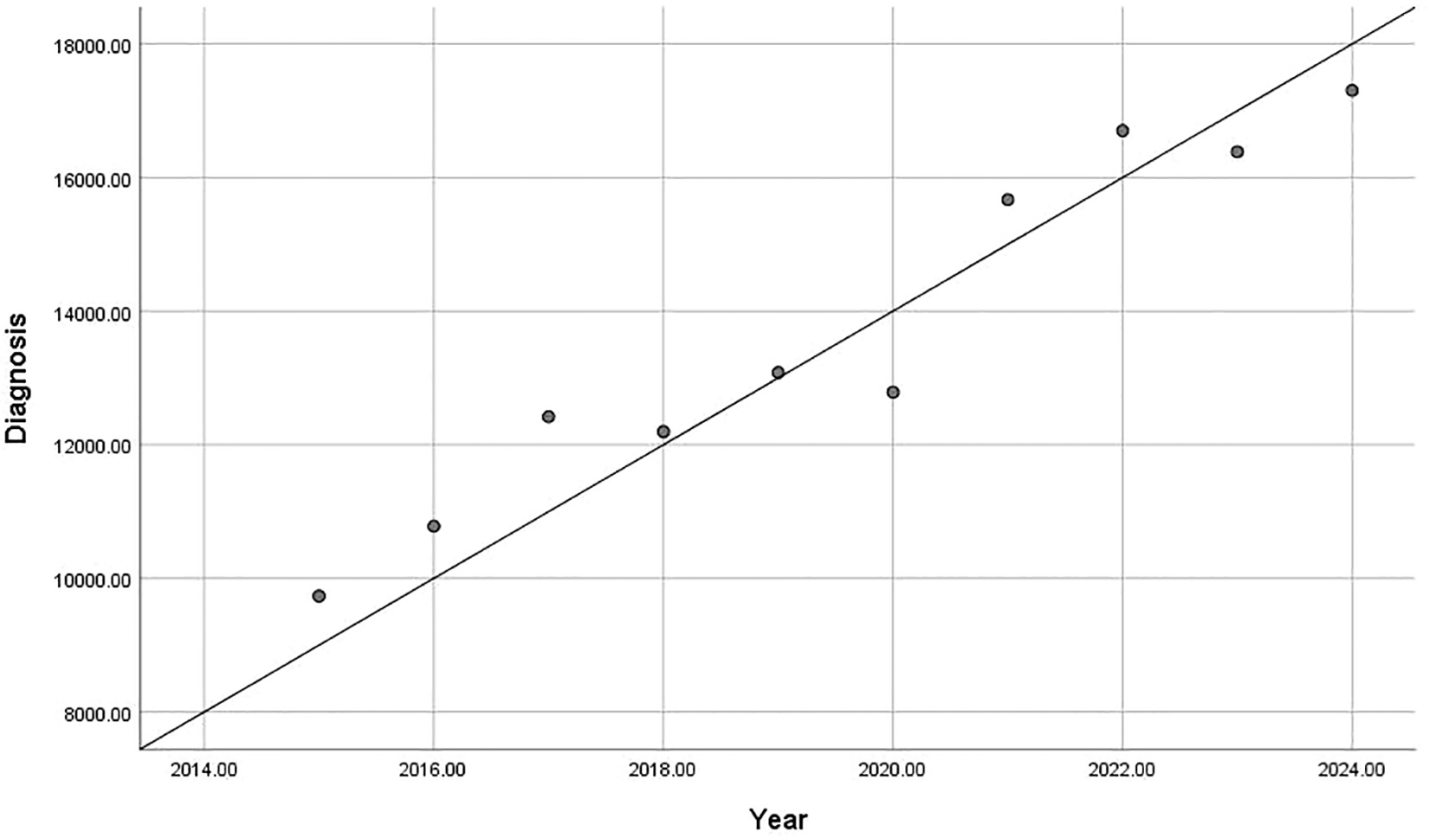
Annual increase in circumcision-related clinic visits over the past decade, based on data from the National Children’s Clinical Research Center (p=0.000, r=0.967).

**Figure 2:**
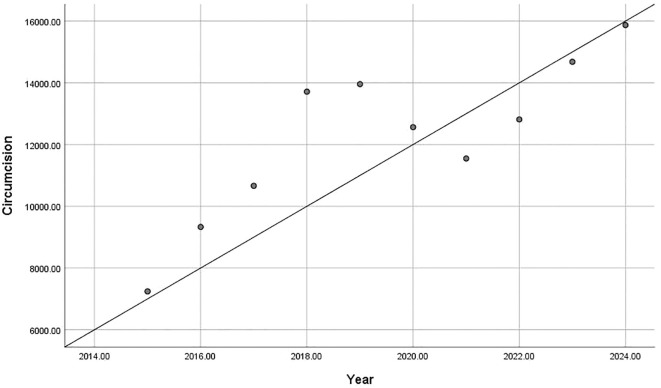
Annual increase in circumcision surgeries over the past decade, based on data from the National Children’s Clinical Research Center (p=0.003, r=0.831).

In recent years, the number of circumcision related clinic visits and surgeries among Chinese children was reported to show an upward trend. According to the National Children’s Clinical Research Center (Chongqing), between 2015 and 2024, the number of circumcision clinic visits showed a significant increased trend (r = 0.967, p < 0.01; **Figure 1**). Similarly, the number of circumcision surgeries increased significantly (r = 0.831, p < 0.01; **Figure 2**). These findings suggest that, despite the high prevalence of physiologic phimosis in childhood, most of which resolves naturally with growth and development surgical intervention is increasingly accepted and even actively chosen by parents.

It is worth noting that the increase in circumcision surgeries is not entirely driven by medical indications. Data from the Children’s Hospital of Chongqing Medical University indicate that, over the past decade, only some circumcision cases met traditional medical indications (e.g., such as pathologic phimosis or recurrent balanoposthitis. A large number of surgeries were performed at the initiative of parents. This phenomenon reflects that parental decision-making regarding circumcision is no longer entirely dependent on medical professional advice, but is influenced by a wider range of informational and sociocultural factors.

### 3.2 Parental decision-making factors

In a questionnaire survey of 56 parents, 64.29% (36/56) expressed a preference for circumcision for their children, indicating a high level of acceptance of the procedure. The key factors influencing parental decision-making can be categorized into facilitating and hindering factors.

#### 3.2.1 Factors promoting surgery

Trust in Internet medical information (OR = 6.054, p = 0.047): parents who exhibited greater trust in internet acquired medical information were significantly more likely to undergo surgery. This finding suggests the substantial influence of online health information dissemination, both in terms of content and delivery on parental decision making.

Long term exposure to recommended surgical content (OR = 7.500, p = 0.015): Parental acceptance of circumcision increased significantly with long term exposure to content about the necessity of surgery (e.g., short video recommendations, parental experience sharing, etc.) on social media or search engines (**Table 1**). This result reflects the critical role of frequency of information exposure in decision making, especially in the context of algorithmic recommendation mechanisms in social media, where parents are prone to develop an “information echo chamber effect”.

**Table 1:** Logistic regression analysis of risk factors influencing parental decision to seek circumcision.

	OR (95% CI)	P-value
Are you worried about surgery?		P=0.044*
No	1	
Yes	0.239 (0.059-0.965)	
Whether or not to trust information on the Internet		P=0.047*
No	1	
Yes	6.054 (1.027-35.683)	
Whether or not the information on the Internet to which you are exposed recommends circumcision		P=0.015*
No	1	
Yes	7.500 (1.481-37.974)	

*p-value < 0.05 indicates a statistically significant.

#### 3.2.2 Factors impeding surgery

Concerns about surgical risks (OR = 0.239, p = 0.044): parents with high perceptions of surgical complications (e.g., scarring, bleeding, infection, insufficient foreskin, etc.) had a significantly lower probability of choosing surgery. This finding suggests that perceived surgical risk serves as a strong deterrent in parental decision-making, and that the way risks are presented in different information channels may influence parental attitudes (**Table 1**).

### 3.3 Internet and media influence

The mode of information dissemination was closely related to parental decision-making, and different media platforms showed significant differences in the dissemination of information about circumcision.

#### 3.3.1 Platform-specific content

Baidu/Academic Database: The content on these platforms is primarily based on medical research and professional guidelines, emphasizing the indications for surgery and postoperative risks (3). Parents searching for information from such sources are usually more inclined to make rational decisions and consider the necessity of surgery.

**Figure 3:**
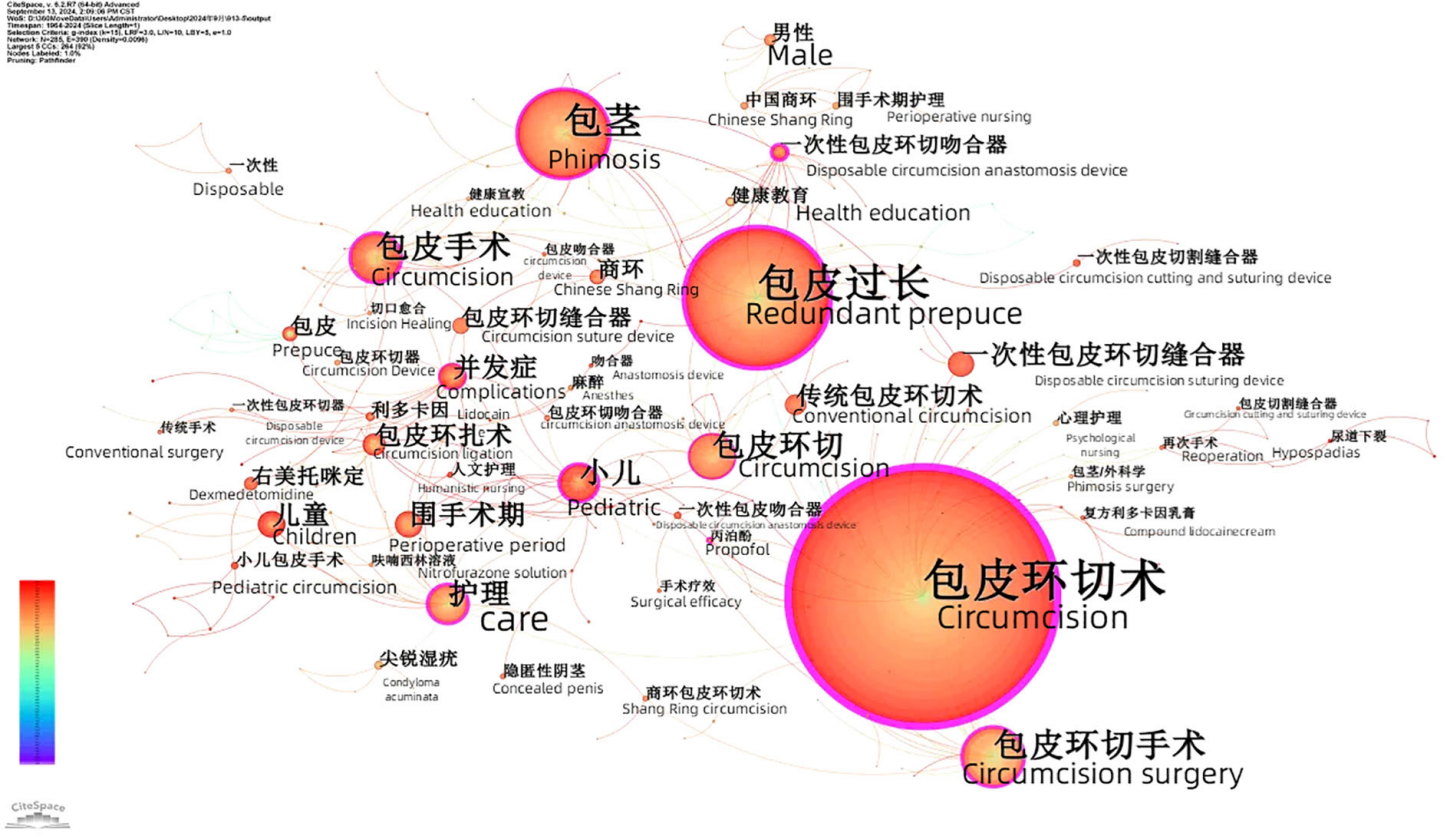
Keyword co-occurrence mapping.

**Figure 4:**
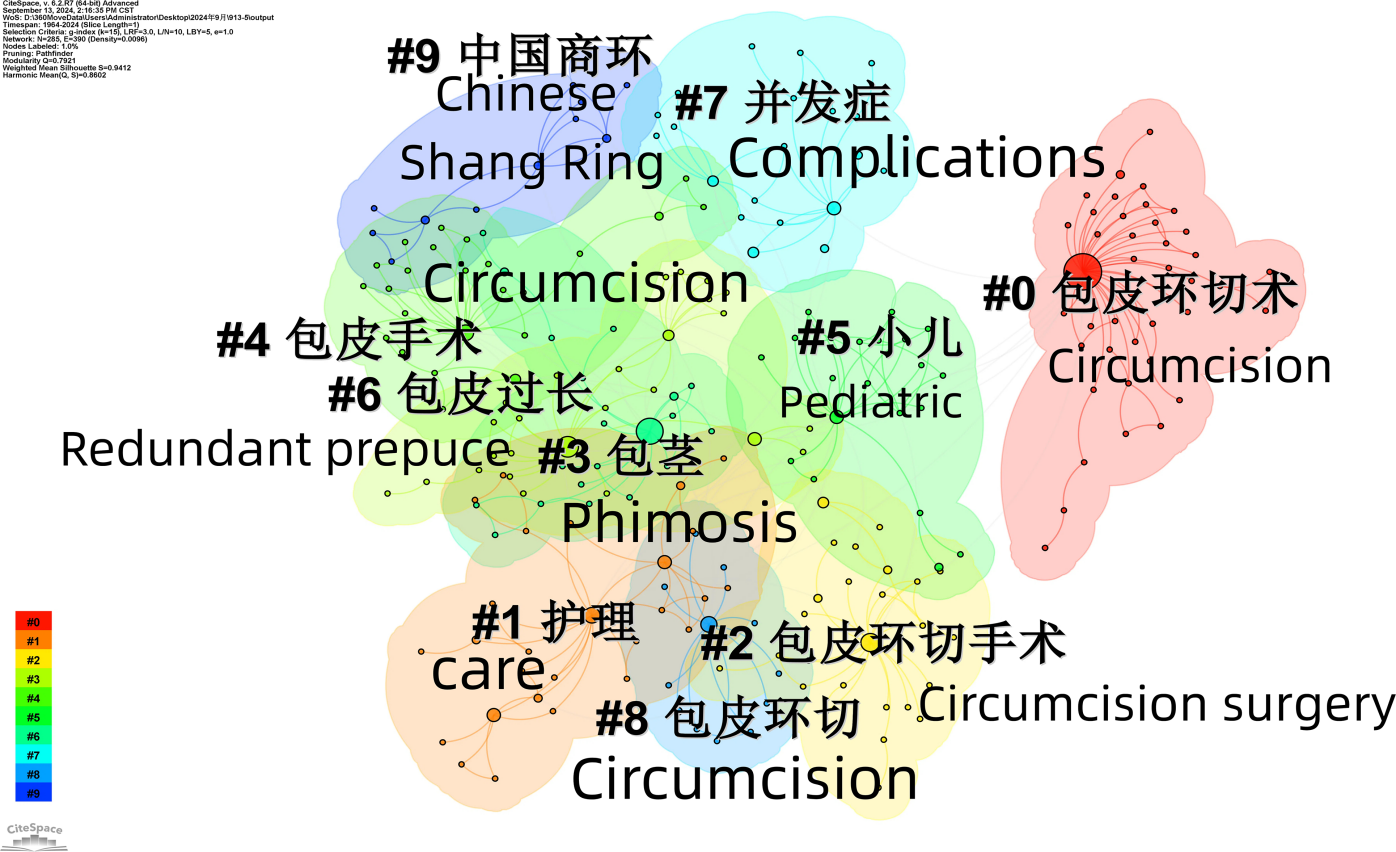
Keyword clustering mapping.

Co-word analysis is one of the most important tools to reveal research hotspots. By analyzing keywords, abstracts and other citation-related data, a visual knowledge map is generated. In this study, a total of 285 high-frequency keywords were extracted, forming 390 connecting lines. The keyword co-occurrence map (**Figures 3**, **4**) shows that “circumcision” is the most dominant node, followed by “phimosis” and “prepuce.” The thickness and density of the connecting lines indicate the strength of keyword co-occurrence, reflecting the degree of association between different keywords. Early studies focused on techniques such as circumcision, clamping, and CO_2_ laser, while recent studies have explored new areas such as day surgery, comprehensive perioperative care, and combined anesthesia. This suggests that future research may increasingly focus on the comprehensive therapeutic efficacy of the procedure and postoperative care.

Keyword cluster analysis provides an intuitive reflection of research hotspots. Based on Cite Space software and the LLR (Log Likelihood Ratio) algorithm, we conducted a cluster analysis of keyword co-occurrence, and the results show that research in this field is predominantly focused on “circumcision”, “nursing” and “circumcision.” The clustering map (**Figure 4**) shows a network density of 0.0096 and a modularity value (Q) of 0.7921, indicating a strong clustering effect. Besides, the average silhouette value (S) is 0.9412, indicating high homogeneity among the clusters. The top five clusters have an average publication year range from 2010 to 2013, indicating that the topics covered in this period have matured. The largest cluster, “circumcision,” contains 42 keywords, mainly related to circumcision, phimosis, phimosis, psychological care, and the use of compound lidocaine cream (**Table 2**).

**Table 2:** Main keywords for clustering.

Position in a ranking of names	Cluster name	Main keywords	Average year	Number of keywords
1	circumcision	Circumcision (34.44, 1.0E-4); Hypospadias (11.88, 0.001); Phimosis (9.44, 0.005); Psychological care (8.92, 0.005); Compound lidocaine cream (8.92, 0.005)	2011	42
2	nursing care	Nursing (28.67, 1.0E-4); Perioperative (20.73, 1.0E-4); Urinary tract infection (9.12, 0.005); Furacilin solution (9.12, 0.005); Topical care (9.12, 0.005)	2011	28
3	circumcision	Circumcision (46.79, 1.0E-4); AIDS (9.63, 0.005); Disposable circumcision anastomosis (9.63, 0.005); Surgical efficacy (9.63, 0.005); Traditional circumcision (6.72, 0.01)	2010	26
4	penile	Circumcision (34.8, 1.0E-4); Disposable circumcision suture (31.41, 1.0E-4); Disposable circumcision anastomosis (13.03, 0.001); Application (8.86, 0.005); Circumcision (6.67, 0.01)	2013	26
5	circumcision	Circumcision (46.06, 1.0E-4); Circumcision anastomosis (10.99, 0.001); Incision healing (10.99, 0.001); Rehabilitation (10.99, 0.001); Circumcision protective pants (10.99, 0.001)	2012	25

Xiaohongshu, a social media focusing on life experience sharing, exhibits significant user engagement in circumcision-related discussions. These discussions, primarily within parents’ groups, cover a broad range of topics through first-level and second-level comments. Users raised a variety of questions and opinions, covering different stages from childhood to adulthood, including the necessity of surgery, the timing of the operation, and post-surgery recovery. A large number of users shared their personal experiences and opinions through comments and replies. This interaction not only increases the popularity of the post but also creates a small community about circumcision. Since parents often play a leading role in making decisions about children’s health, we hypothesized that the experiences they shared on this platform could provide insights to other parents in informed decision-making.

#### 3.3.2 Opinion bias and information visibility bias (sentiment analysis)

In this study, the top ten posts related to circumcision on the Xiaohongshu platform were selected as the research objects. The study mainly focuses on the physiological effects (sensitivity, hardness), aesthetic effects, and the credibility of medical information provided to parents. Among them, the post with the highest engagement stands out: it received 14,000 comments, 91,000 likes, 137,000 shares, and 33,000 favorites, which indicates significant social attention and influence within the young parent community. The data collection system was constructed using the Python programming language, and structured data from the comment section was obtained by reverse parsing the encrypted parameters of the platform through Node.js. Sentiment analysis was performed using the SnowNLP module to establish the following classification criteria:

Negative Sentiment: (0, 0.5), Neutral Sentiment: = 0.5, Positive Sentiment: (0.5, 1).

During the data cleaning process, emoticons, irrelevant characters, and off-topic content (e.g., “What’s a circumcision ring?” “What do I do?” etc.) were excluded. A total of 7112 valid primary and secondary comments were obtained.

##### 3.3.2.1 Sentiment distribution characteristics first-level comment analysis (n=2857)

Negative sentiments: 34.48%(985/2857), neutral sentiments:9.70% (277/2857), positive sentiments: 55.82% (1595/2857).

##### 3.3.2.2 Secondary comment analysis (n=4255)

Negative sentiments: 31.89%(1357/4255), neutral sentiments:9.99% (425/4255), positive sentiments: 58.12% (2473/4255).

Overall emotional disposition:

The percentage of comments with a significant affective tendency (≠0.5) was 90.13% (6410/7112). Among these, 57.20% (4068/7112) of the total sample comments were positive, 32.93% (2342/7112) were negative, and 9.87% (702/7112) were neutral comments.

Analysis of the resultant data showed that platform users’ attitudes toward child circumcision exhibited significant emotional differentiation:

Dominant Positive Tendency: More than half of the comments (55.82% of primary comments and 58.12% of secondary comments) expressed a supportive attitude, echoing the rising trend of clinical procedures. This trend may be attributed to parents’ recognition of “preventive medicine,” the effect of shared success stories, and the platform’s algorithm which prioritizes the display of positive content.

Persistent Negative Views: About 32.93% of the comments were negative. These concerns primarily focused on perceived surgical risks (anesthesia safety, postoperative complications), the questioning of non-necessary medical interventions, and conflicting cultural traditions and medical norms.

Decision-making uncertainty in the neutral group: About 9.87% of comments did not explicitly take a stance, indicating that some parents are still at the information assessment stage. This suggests a need to enhance the accessibility of specialized medical information.

## 4 Discussion

### 4.1 Influences on parental decision-making

Parental decision-making about child circumcision is influenced by multiple factors, which can be categorized into three main groups: (1) medical risk perceptions; (2) socioeconomic factors; and (3) psychological cognitive biases. These factors work together to shape parental attitudes and determine their decisions regarding circumcision.

#### 4.1.1 Medical risk factors

Medical risks are one of the most immediate concerns for parents, mainly including surgical complications and anesthesia risks. Although modern surgical techniques have significantly reduced the risks related to circumcision, parents may still be concerned about possible side effects of the procedure.

Common postoperative complications include infection, bleeding, postoperative pain, scarring, and urethral stricture (1). Although this surgery is usually considered a low-risk surgery, some parents remain cautious due to possible postoperative recovery issues.

Anesthesia, whether general or local, may trigger allergic reactions, respiratory depression, and other adverse effects (4). Although such risks are extremely rare, parental concerns about anesthesia safety may influence their decision to refuse surgery.

#### 4.1.2 Socioeconomic factors

Socioeconomic status plays an important role in medical decision-making, and the cost and recovery period of circumcision may pose a greater challenge for low-income families.

Financial burden: Although Medicare-covered surgery is available in public hospitals in some areas, surgery costs can be significantly higher in the private sector, especially for elective surgeries (3). As a result, low-income families may be hesitant about surgery.

Time investment: The recovery from surgery usually takes days to weeks, which may disrupt a child’s schooling or the family’s daily routines. Parents need to weigh the short-term inconvenience of recovery against the potential long-term health benefits of the procedure.

#### 4.1.3 Psychological cognitive bias

Parents’ perception of risk and the way they receive information may lead to an overestimation of the necessity for surgery and an underestimation of the likelihood of natural remission of a physiologic prepuce. This bias is closely related to the sociocultural tendency toward “preventive medicine”.

Risk magnification effect: Some parents believe that not undergoing circumcision may lead to urinary tract infections, reproductive health issues, and other complications. However, under normal circumstances, the foreskin covering the glans does not pose significant health risks (3).

Herd mentality: Parents may be influenced by others to adopt the belief that “the majority choice of surgery is the right choice,” a socially normative perception that may undermine rational decision-making.

#### 4.1.4 Behavioral science explanations for surgical choice

Parents’ decision to opt for unnecessary surgical procedures can be understood through behavioral science theories. On one hand, this phenomenon relates to cognitive biases (5), while medical decisions should ideally be based on an accurate understanding of potential risks, parental risk perception is often distorted by cognitive biases and irrelevant information (6). On the other hand, the principle of risk aversion suggests that when confronted with uncertain future outcomes, parents may prefer to accept a certain, immediate cost (e.g., the surgery) to avoid a perceived, larger future risk (e.g., reproductive health issues) (7). Social media content may exploit this psychological tendency by amplifying the perceived risks associated with not undergoing circumcision, thereby aligning the choice of surgery with parents’ protective instincts.

### 4.2 Media’s triple mechanism of influence

The role of social media in parental healthcare decision-making is becoming increasingly important. The methods of information dissemination, content recommendation algorithms, and modes of social interaction may shape parents’ perceptions of circumcision. Research has found that social media influences parental decision-making through a threefold mechanism.

#### 4.2.1 Algorithmic visibility bias

The platform’s recommendation algorithm favors content with high interaction rates, leading to the dominance of specific views in the information stream. This study found that pro-surgery comments on the Xiaohongshu platform were more frequently exposed than opposing views. The interactivity of posts amplifies the effect, as the sharing of surgical experiences and before-and-after comparison photos holds stronger visual appeal and emotional resonance, making them more likely to be liked and shared.

As a result, the information becomes skewed, and platform algorithms may inadvertently amplify particular points of view, exposing users to more homogenous information and overshadowing more objective medical advice.

#### 4.2.2 Group polarization effect (echo chambers)

When parents view relevant content on social media, they are more likely to join discussion groups that share similar views, leading to a polarization of group opinions. This phenomenon, known as the “opinion vortex,” is particularly evident on social media platforms such as Xiaohongshu. The pro-surgery group is more active, with more than half of the popular posts analyzed in this study being pro-surgery. This is nearly 30% higher than the percentage of surgery recommendations outlined in medical guidelines. While negative comments about surgery account for around 28% of the responses, they have a relatively low reach and interaction, weakening their influence on parental decision-making.

#### 4.2.3 Information cocoon enhancement

The study found that when parents search for circumcision information on social media, the algorithm tends to push similar content, creating an “information cocoon” phenomenon. As the algorithm continues to push content aligned with their initial perceptions, parents are less likely to encounter contrary viewpoints. Longer exposure to a single perspective can strengthen their decision-making stance and amplify cognitive biases, even when these views diverge from professional medical guidelines (8).

### 4.3 Theoretical applications of knowledge and power

Foucault’s (2001) theory of “knowledge-power” can provide an explanatory framework for this study. In the digital age, medical discourse is not only controlled by professional organizations, but also influenced by social media, commercial capital, and the sharing of personal experiences, leading to the construction of circumcision as a “normative health practice.”

#### 4.3.1 Penetration of institutional authority

Health-care organizations have partnered with popular science bloggers to popularize the necessity of surgery through short videos and social media, integrating surgery into the concept of “scientific legitimacy.” Surgery videos posted by doctor accounts on platforms such as Shakeology are often highly credible, making parents more receptive to surgical recommendations.

#### 4.3.2 Capital-driven narratives

Commercialized platforms, such as Baidu’s bidding rankings, may prioritize content from healthcare providers with a profit-making agenda, which can lead to the overgeneralization of the procedure’s indications. Some hospitals exaggerate the necessity of circumcision in their marketing, leading parents to perceive it as a necessary medical practice (9).

#### 4.3.3 Dissolution of parental subjectivity

This study found that 78% of the parents interviewed stated they “follow the advice of Netflix doctors,” reflecting how individual decision-making is gradually being hostage to systemic discourse in the age of social media. This phenomenon shows a profound change in power relations in the dissemination of medical knowledge in the Internet era.

### 4.4 Responsibility for web-based popularization of science

Multidimensional interventions are needed to improve the quality of medical decision-making.

#### 4.4.1 Platform governance

Labeling of sources: Social media platforms should be required to label medical content with sources, such as “certified by a tertiary hospital” or “recommended by an authoritative medical guide,” to increase the credibility of the information. Drawing on (10) approach to HIV popularization, this measure would help reduce misleading labels for non-professional users and limit the sharing of non-professional experiences.

#### 4.4.2 Parental digital literacy

Providers can introduce visualization tools (e.g., risk/benefit comparison modules for surgery) and develop decision aids to help parents make more scientific decisions (2).

#### 4.4.3 Policy regulation

It is crucial to strengthen the regulation of misleading information by including misleading medical propaganda under the scope of penalties outlined in the Advertising Law, and adhere to the strict indication criteria for male circumcision as recommended by the WHO (4).

This study reveals two central paradoxes in the decision-making process of Chinese parents regarding circumcision for their children aged 6–12 years: (1) information overload is inversely related to the quality of decision-making. Although the Internet provides unprecedented access to medical information, algorithmic recommendation mechanisms (e.g., likes and dislikes, personalized tweets) led parents to rely more on lay sources (e.g., social media experience sharing) than on clinical guidelines. This “visibility bias” increases the exposure of emotional and polarizing content, creating an information cocoon that ultimately undermines rational decision-making. Despite the vast amount of information available, parents found themselves in the paradox of “the more they searched, the more anxious they became” due to cognitive biases (e.g., confirmation bias, herd effect), and the quality of their decision-making did not improve with the amount of information; and (2) there is a conflict between medical norms and social discourse. International clinical guidelines generally recommend conservative treatment for physiological prepuce (1), but online narratives - through short videos and user comments - have framed the surgery as a “standard of scientific parenting” and even positioned it as a preventive health measure. This discursive power permeates through the media platforms, allowing parents to view surgery as a ‘necessary option’ rather than one based on individual medical assessment. A Foucaultian analysis of power shows that capital-driven medical marketing, algorithmic recommendation mechanisms, and parental risk aversion reinforce this trend.

The novelty of this study lies in its interdisciplinary integration of medical science and communication studies, demonstrating how digital media ecosystems directly influence decision-making regarding a specific pediatric surgical procedure in China. This research provides a critical theoretical foundation for enhancing the quality of online health information and fostering a more authoritative medical communication environment.

This study has several limitations. Most notably, the generalizability of the quantitative findings from the parent questionnaire is limited by the relatively small sample size. Although the mixed-methods approach provides rich contextual insights, the statistical power of the regression models is constrained. Consequently, the identified risk factors should be regarded as preliminary hypotheses, necessitating future large-scale, multi-center, and nationally representative surveys to validate these associations and establish more stable effect estimates. Secondly, the cross-sectional survey design employed in this study, while capable of revealing associations between factors, cannot establish the direction of causality. Therefore, future research should focus on three points: (1) Policy intervention: establish a quality certification system for online medical popularization, limit the dissemination of misleading content, and encourage authoritative organizations (e.g., pediatric medical societies) to engage with social platforms to implement the strategy of “replacing authoritative sources”; (2) Clinical practice: strengthen doctor-patient communication and correct parents’ perceptions through decision-making tools (e.g., visualization charts); (3) Expansion of the study: this study’s limitations, particularly its reliance on single-center data, call for future research that incorporates urban-rural and economic stratification and tracks the long-term effects of policy interventions. The absence of publicly available, systematic national statistics on circumcision rates in China underscores the critical need to establish such databases to facilitate more robust epidemiological surveillance and evidence-based policy formulation.

## 5 Conclusion

In summary, the solution to this socio-medical problem requires a collaborative effort from multiple parties to restructure the normative framework of health communication while safeguarding the freedom of information, in order to achieve a balance between science and autonomy in parental decision-making.

## Data Availability

The original contributions presented in the study are included in the article/supplementary material. Further inquiries can be directed to the corresponding authors.
